# Registry-Dependent Potential for Interfaces of Gold with Graphitic Systems

**DOI:** 10.1021/acs.jctc.1c00622

**Published:** 2021-10-29

**Authors:** Wengen Ouyang, Oded Hod, Roberto Guerra

**Affiliations:** † Department of Engineering Mechanics, School of Civil Engineering, Wuhan University, Wuhan, Hubei 430072, China; ‡ Department of Physical Chemistry, School of Chemistry, The Raymond and Beverly Sackler Faculty of Exact Sciences and The Sackler Center for Computational Molecular and Materials Science, Tel Aviv University, Tel Aviv 6997801, Israel; § Center for Complexity and Biosystems, Department of Physics, University of Milan, 20133 Milan, Italy

## Abstract

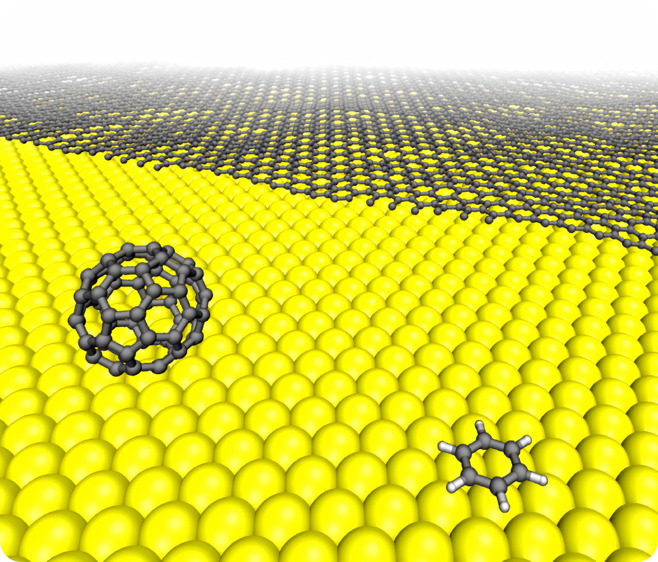

We present a semi-anisotropic interfacial potential (SAIP) designed to classically describe the interaction between gold and two-dimensional (2D) carbon allotropes such as graphene, fullerenes, or hydrocarbon molecules. The potential is able to accurately reproduce dispersion-corrected density functional theory (DFT+D3) calculations performed over selected configurations: a flat graphene sheet, a benzene molecule, and a C_60_ fullerene, physisorbed on the Au(111) surface. The effects of bending and hydrogen passivation on the potential terms are discussed. The presented SAIP provides a noticeable improvement in the state-of-the-art description of Au–C interfaces. Furthermore, its functional form is suitable to describe the interfacial interaction between other 2D and bulk materials.

## 1 Introduction

The reproducibility of the phenomena emerging at the interface between two bodies in contact is often limited by the availability of clean and well-controllable surfaces. Gold and graphene are two very stable materials that can be produced with a high level of crystallinity and cleanness over large surface areas. For these reasons, the gold–graphene interface has been the prototypical choice for a large number of case studies, including diffusing and sliding gold nanoclusters,^1−6^ nanomanipulation of graphene nanoribbons,^7−9^ plasmon-enhanced optics,^10,11^ sensing and biomedical applications,^12^ and surface-enhanced Raman scattering,^13,14^ among many others.

Despite the vast interest expressed by the scientific community in this composite system, to date, no reliable classical force field is available for computational simulations involving interfaces of gold with graphitic systems. Since the interaction between graphene and metals is mainly governed by van der Waals forces,^15,16^ a simple two-body Lennard-Jones (LJ) potential, often expressed in the form *V*_LJ_(*r*) = 4ϵ[(σ/*r*)^12^ – (σ/*r*)^6^], has been so far employed and parametrized in order to fit observations from specific experimental setups.^6,7^ However, the oversimplified nature of the LJ potential, with just ϵ and σ setting the two-body dissociation energy and equilibrium length, respectively, makes it extremely challenging to fit more than a few among the several physical quantities impacting the static and dynamical properties of a gold–graphene assembly. As a consequence, previous studies have employed very different parametrizations to match some particular quantities of interest, with ϵ varying from 2.5 to 90.0 meV and σ varying from 2.5 to 3.2 Å,^1,2,6,7,17^ making a straightforward comparison among these studies often impractical.

Beside quantitative errors, even a qualitative description in LJ terms is questionable. For a single gold atom residing on a graphene surface, a typical pairwise isotropic potential (i.e., depending only on the distance between pairs of atoms), such as the LJ potential, would find the minimum energy position, where the gold atom is located over a graphene hexagon center (hollow position). Nevertheless, experiments and first-principle calculations have demonstrated that the atop position, where the Au atom resides over a C atom, is energetically favorable.^18,19^ This exemplifies the need for an anisotropic potential to describe the gold–graphene interfacial energy landscape.

Beyond the single-atom contact, when an extended gold–graphene interface is considered, the sliding energy landscape becomes much more complex due to the intrinsic incommensurability of the contact. In fact, nanoscale graphene flakes and ribbons residing over the (111) surface of gold can be found in both the epitaxially aligned R0 and the R30 tilted orientations.^7,20^ Extended contacts, on the other hand, were predicted to prefer intermediate misfit angles 0 < θ < 30°.^21^ This, in turn, is expected to be manifested in the interfacial tribological properties, where the combination of the weak Au–C interactions and the strong internal cohesive forces of gold and graphene may lead to exceptionally low friction coefficients (<10^–3^)—a condition often referred to as structural superlubricity.^6,22,23^ In superlubric systems, the corrugation energy (CE)—the energy barrier resisting sliding—can decrease to values much below meV per interface atom. Therefore, studies aiming at describing superlubricity, often considering a combination of metals and graphitic materials,^3,7−9,24−26^ are critically sensitive to the chosen experimental or theoretical setup.

It is, therefore, evident that there is an urgent need to develop a new force field able to reliably describe the interaction between graphene and gold. Unfortunately, there is only a handful of experimental studies providing data that can be directly employed to parametrize such a force field. For example, Torres et al. measured an adhesion energy (AE) *E*_a_ = 7687.1 mJ/m^2^ in the case of graphene-covered gold nanoparticles,^27^ while a pull-off force of *F*_a_ = 45.7 ± 5.1 nN was measured by Li et al. for gold-coated atomic force microscopy probes forming a ∼200 nm^2^ contact with a graphite substrate.^23^ Other experiments provide only indirect information based on empirically fitted models or simulations.^6,7,9,28^ The comparison with experiments is further complicated by the presence of surface reconstruction at the gold surface, usually neglected in first-principles calculations due to the large supercell size required to encompass its long (∼6.3 nm) wavelength. Leaving out such reconstruction effects potentially overestimates the computed interaction energies, but in most cases, this approximation yields only minor structural modifications in the model systems.^29^

Due to the lack of direct experimental measurements and accurate computational reference data for the adhesion and corrugation energies of graphene–gold interfaces, we performed dispersion corrected density functional theory (DFT+D3) calculations on the R30 tilted graphene–gold heterojunction. This reference dataset allowed us to parametrize a newly developed semi-anisotropic interfacial potential (SAIP) that is able to simultaneously reproduce the DFT+D3 AE curves and sliding potential energy surfaces (PES). The developed SAIP and its suggested parameterization present a significant advancement with respect to the present stand of classical description of the interfacial interactions in gold and graphene junctions. Furthermore, the SAIP formulation provides a general tool for describing interfaces formed between two-dimensional (2D) materials and bulk solids.

## 2 Potential Description

The presented SAIP is based on the concept of anisotropic interlayer potentials for 2D materials.^30−34^ The potential consists of two terms: an isotropic term that describes the long-range attractive dispersive interactions and an anisotropic term that describes the Pauli-type repulsion between the graphene π electrons and the gold surface electron density. The dispersive term treats long-range van der Waals interactions via a *C*_6_/*r*^6^ LJ type potential, dampened in the short range with a Fermi–Dirac-type function similar to that introduced in dispersion-corrected DFT calculations to avoid double counting of correlation effects^35^
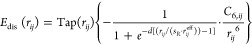
1Here, *r*_*ij*_ is the distance between carbon or hydrogen atom *i* and gold atom *j*, *d* and *s*_R_ are unit–less parameters determining the steepness and onset of the short-range Fermi-type dampening function, and *r*_*ij*_^eff^ and *C*_6,*ij*_ are the sum of effective atomic radii and the pair-wise dispersion coefficients, respectively. The Tap(*r*_*ij*_) function provides a continuous (up to 3rd derivative) long-range cutoff at *r*_*ij*_ = *R*_cut_ to the potential aiming to reduce computational burden^36^
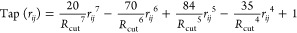
2

**Figure 1:**
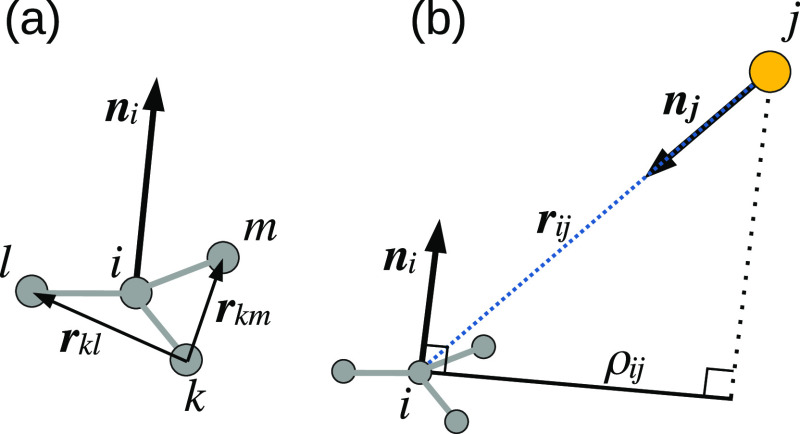
Normal vectors and transverse distance—(a) construction of a normal vector *n*_*i*_ associated with carbon or hydrogen atom *i* (see text); (b) scheme of the relation between the normal vector *n*_*i*_ and the transverse distance ρ_*ij*_ between atom *i* and gold atom *j*.

Following the Kolmogorov–Crespi^30^ scheme, the anisotropic term of the potential is constructed from a Morse-like exponential isotropic term, multiplied by an anisotropic correction, in which the orientation of graphene is described by normal vectors associated with each carbon or hydrogen atom
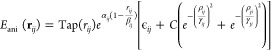
3Here, Tap(*r*_*ij*_) is the cutoff smoothing function of eq 2, α_*ij*_ and β_*ij*_ set the slope and range of the potential, and γ_*ij*_ sets the width of the Gaussian decay factors in the anisotropic correction term and thus determines the sensitivity to the transverse distance, ρ_*ij*_, between carbon or hydrogen atom *i* and gold atom *j* (see Figure 1). *C* and ϵ_*ij*_ are constant scaling factors bearing units of energy. The normalized normal vectors ***n***_*i*_ (i.e., ∥***n***_*i*_∥ = 1) serve to calculate the transverse distance ρ_*ij*_ between pairs of carbon or hydrogen atom (*i*) and gold atom (*j*)
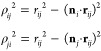
4

Each normal vector ***n***_*i*_ defines the local normal direction to the graphene sheet (or to the benzene molecule) at the position of atom *i* (see Figure 1). Note that the definition of the normal vector is not unique and can follow different schemes.^30^ For example, one can calculate the normal vector of atom *i* by averaging the three normalized cross products of the vectors connecting atom *i* to its three nearest neighbors *k*, *l*, and *m* or, in a more simple way, by calculating it as ***n***_*i*_ = (***r***_*kl*_ × ***r***_*km*_)/(∥***r***_*kl*_∥ ∥***r***_*km*_∥) [see Figure 1a]. In our implementation, the former definition is adopted. However, when dealing with a flat graphene (or benzene molecule) lying on the *xy* plane, all definitions of the normal vector should give **n**_*i*_ = *ẑ*, making the specific choice irrelevant. In the case of curved graphene, such as for nanotubes, all normal vector definitions should produce very similar results except for extremely small radii of curvature.^30,37^

To account for the isotropic nature of the isolated gold atom electron cloud, their corresponding normal vectors are assumed to lie along the interatomic vector ***r***_*ij*_. Notably, this assumption is suitable for many bulk material surfaces, for example, for systems possessing s-type valence orbitals or metallic surfaces, whose valence electrons are mostly delocalized, such that their Pauli repulsions with the electrons of adjacent surfaces are isotropic. Caution should be used in the case of very small gold contacts, for example, nanoclusters, where edge effects may become relevant.

Following the above assumption, ***n***_*j*_∥***r***_*ij*_, one gets ρ_*ji*_ = 0. The latter simplification reduces the anisotropic term to the following final form
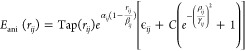
5

## 3 Model Systems and Methods

### 3.1 Model Systems

**Figure 2:**
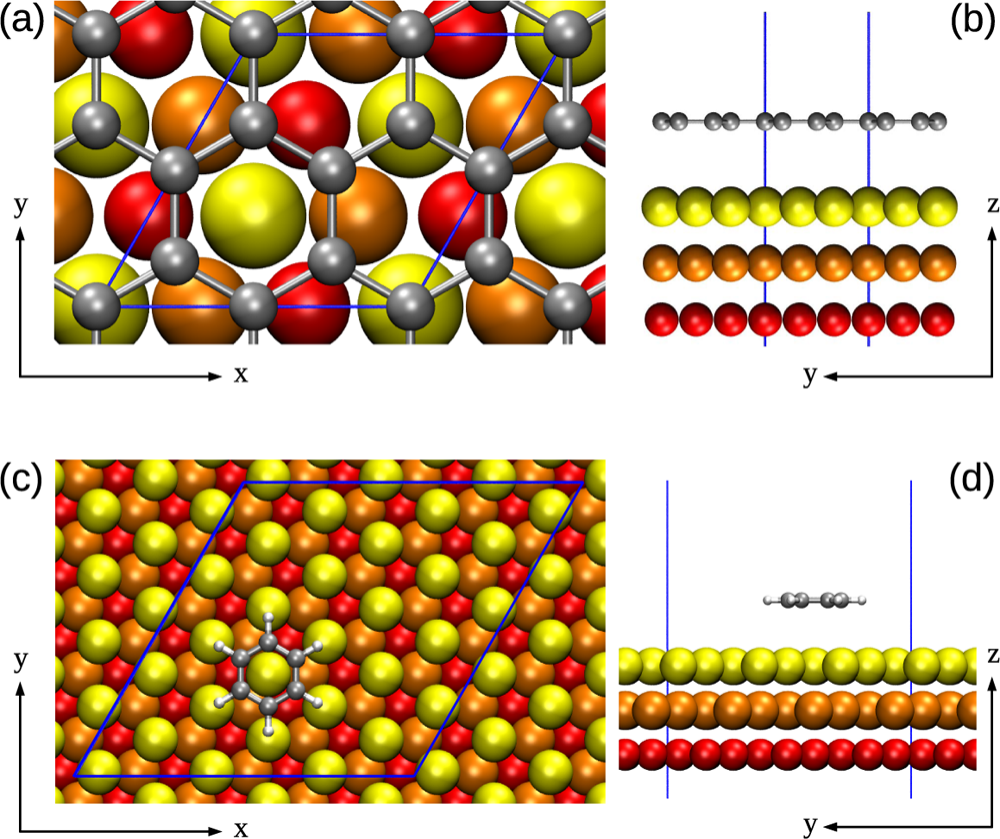
Reference model systems—(a) top and (b) side views of the graphene/Au(111) model system in the “atop” configuration. (c) Top and (d) side views of the benzene/Au(111) model system in the “hollow” configuration. Carbon and hydrogen atoms are depicted in gray and white, respectively. First, second, and third Au layers are colored in yellow, orange, and red, respectively. Blue lines outline the primitive cell.

As model systems for the parameterization process, we choose the two interfaces depicted in Figure 2. The first is composed of a graphene layer positioned at the R30 stacking configuration over three Au(111) layers.^20^ The second is composed of a benzene molecule residing over three Au(111) layers. DFT spin-polarized calculations were performed by the Perdew–Burke–Ernzerhof (PBE) exchange–correlation functional within the generalized gradient approximation augmented by Grimme’s D3 long-range dispersion correction and the Rappe–Rabe–Kaxiras–Joannopoulos ultrasoft core-corrected pseudopotentials, as implemented in the Quantum ESPRESSO software.^38−41^ Previous benchmark DFT calculations of metal–organic frameworks indicate that the PBE exchange correlation density functional approximation with the D3 dispersion correction can accurately describe the energetics of complex systems involving organic and inorganic components.^39,42,43^ However, whether this is the best DFT approach (out of tens of currently available dispersion-oriented density functional approximations) for the system under consideration cannot currently be concluded mainly due to the lack of experimental results or high-accuracy computational reference data.

Kinetic-energy cutoffs of 600 eV for the wavefunctions and 4952 eV for the density were employed, with a reciprocal-space mesh of 7 × 7 × 1 *k*-points for the Au(111)–graphene interface and 3 × 3 × 1 *k*-points for the Au(111)/benzene system (see below). Convergence of total energy over the above cutoffs and *k*-points grid was established. Periodic boundary conditions were applied in all directions. In all the calculations, graphene or benzene were kept flat^15^ and the gold atoms were maintained in the fcc bulk configuration.

In the Au(111)–graphene case, the lateral stress was minimized by iteratively rescaling the system size. The final supercell vector length and angles were *a* = *b* = 4.965638 Å, *c* = 30 Å, α = β = 90°, and γ = 60° (see Figure 2a,b). The out-of-plane periodicity *c* was chosen to be as large as possible to avoid interactions with replicas along the vertical direction. The calculated lattice constants of the isolated bulk gold and graphene systems were 2.8825 and 2.4652 Å, corresponding to a final strain of −0.54 and +0.7%, respectively, in the relaxed composite system. In the Au(111)/benzene and Au(111)/C_60_ cases, we have employed a supercell with *a* = *b* = 14.896914 Å, *c* = 30 Å, α = β = 90°, and γ = 60° (see Figure 2c,d). Equilibrium distances of 3.5 Å between graphene and gold, 3.3 Å between benzene and gold, and 3.3 Å between C_60_ and gold were found by rigid vertical displacement of the adsorbate molecule.

### 3.2 Fitting Protocol

**Figure 3:**
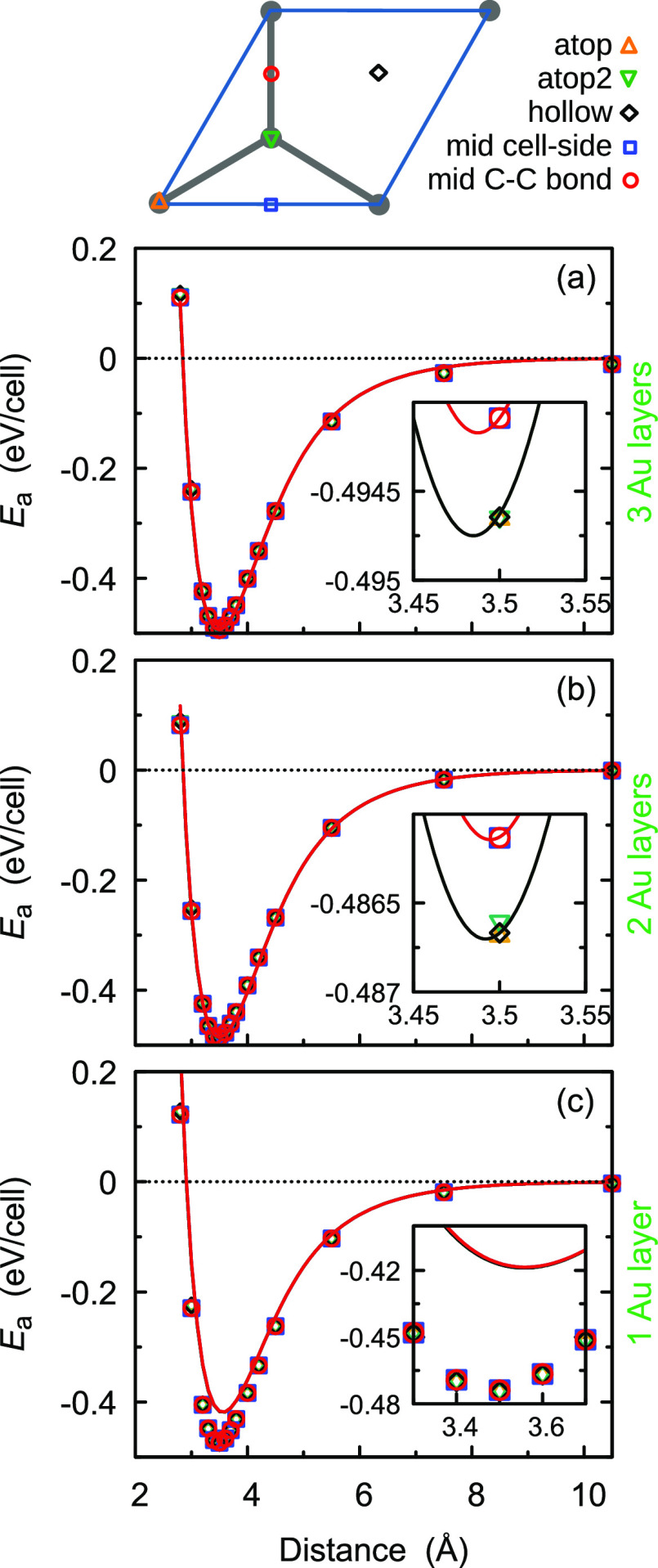
Graphene on gold: adhesion energy curves of the graphene–gold heterostructure with (a) trilayer gold, (b) bilayer gold, and (c) single layer gold. Symbols and lines represent DFT+D3 reference data and SAIP results, respectively. Insets provide a magnification around the minimum energy. Different symbols and colors represent different stacking modes, as depicted in the top panel (see text).

The parameters of the SAIP were determined against reference *M* = *M*_b_ + *M*_s_ DFT datasets including *M*_b_ AE curves and *M*_s_ sliding PESs. The AE curves were calculated for five high-symmetry stacking modes (see top panel of Figure 3), which are concisely denoted by ***r***_*m*_, where *m* ∈ [1, *M*_b_], such that  and *N*_*m*_ is the number of atoms in configuration *m*. Each AE curve includes 15 data points as a function of the gold–graphene (gold–benzene, or gold–fullerene) distance. The sliding PESs were obtained at a fixed vertical (*z*) distance of 3.5 Å, by rigidly shifting the adsorbate along the lateral (*x*–*y*) directions with respect to the gold substrate. The single-point total energy at each of the 441 points of a uniform mesh grid was recorded. The origin (0,0) configurations of the PESs correspond to those presented in Figure 2.

Optimal SAIP parameters were obtained by minimizing the following objective function that quantifies the difference between the DFT reference data and the potential predictions
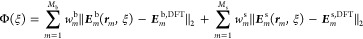
6Here, ∥·∥_2_ is the Euclidean norm (2-norm) that measures the difference between the SAIP predictions and the DFT reference data, ξ represents the set of potential parameters, and ***E***_*m*_^b^(***r***_*m*_,ξ) and ***E***_*m*_^s^(***r***_*m*_,ξ) represent the *M*_b_ AE curves and *M*_s_ sliding PES data sets, respectively. *w*_*m*_^b^ and *w*_*m*_^s^ are the corresponding weighting coefficients. The reference DFT interfacial energies, ***E***_*m*_^b,DFT^ and ***E***_*m*_^s,DFT^, are obtained as follows: for any given configuration *m* of the heterostructure, the total energy is first obtained from DFT+D3 calculation: ***E***_*m*_^DFT,total^. Then, the energies of the isolated graphene, ***E***_*m*_^DFT,graphene^, and of isolated gold, ***E***_*m*_^DFT,gold^, are computed separately using the same cell as that of the composite system. The DFT interfacial energy appearing in eq 6 is then defined as
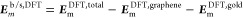
7

Since the CE—defined as the difference between maximum and minimum PES energy—is much smaller than the AE (∼1 vs hundreds of meV/cell), the energy weights for the AE curves were set to *w*_*m*_^b^ = 1 (*m* = 1, ..., 5), and those of the sliding PESs were chosen as , thus providing comparable precision. The optimization was carried out using MATLAB with an interior-point algorithm^44,45^ (further details are provided in refs (34) and (37)). To obtain transferable parameters that can account for varying gold thickness, we first parameterized the potential for the heterostructure with trilayer gold using the objective function defined in eq 6; then, we added the training sets of the heterostructure with bilayer and single layer gold and reparameterized the potential following the same procedure.

## 4 Results and Discussion

The fitted parameters for Au–C and Au–H interactions are reported in Table 1. We note that the negative sign in the *C* parameter can be attributed to the fact that the atop position of an Au atom on graphene is energetically favorable with respect to the hollow position,^18,19^ where the Au atom resides over the graphene hexagon center (see top panel of Figure 3). When applying this potential to describe the interfacial interaction in other 2D and bulk material interfaces, the parameter *C* can be either positive or negative, depending on the sliding energetics.^30,37^

**Table 1:** Potential Parameters—List of SAIP (Equations 1, 3, and 5) Parameters for the Interfacial Au–C and Au–H Interactions

atom pair	α	β (Å)	γ (Å)	ϵ (meV)	*C* (meV)	*d*	*s*_R_	*r*_eff_ (Å)	*C*_6_ (eV Å^6^)
Au–C	13.56556	3.69133	1.01755	7.09648	–1.03683	11.05865	1.06356	3.75526	81.58471
Au–H	4.30650	3.78996	10.68118	225.08878	–111.68915	18.61492	0.983319	3.35076	70.68654

### 4.1 Graphene on Gold

Figure 3 shows the comparison between the AE curves of the Au(111)–graphene heterostructure with trilayer (Figure 3a), bilayer (Figure 3b), and single layer gold (Figure 3c) obtained using DFT+D3 (symbols) and the SAIP using the parameters provided in Table 1 (solid lines), calculated at different stacking modes. Good agreement between the DFT and SAIP is obtained especially in the tri- and bilayer case, while for the single layer, a larger discrepancy is found. Note that the DFT calculations show a similar AE, regardless of the number of gold layers, while the SAIP predicts a reduced adhesion in the case of a single gold layer. We associate the former with the fact that decreasing the number of layers reduces the adhesive interactions, but at the same time the undercoordinated Au surface becomes more chemically reactive. This is also related to the known increased atomic density of gold surfaces with respect to bulk.^46−49^ Such compensation yields an almost unchanged AE value, just slightly reduced with respect to the bilayer case. This change of reactivity is lacking in the present form of the SAIP. Here, the reduced adhesion obtained in the case of the gold monolayer is clearly due to the reduced number of Au–C interacting pairs, whereas the binding energies obtained for the 2- and 3-layer substrates are very similar being the third layer distant from the graphene surface. The average deviations, , for the AE curves of the tri-, bi-, and single-layer Au(111) systems are 11.38, 11.43, and 70.95 meV/cell, respectively.

As mentioned earlier, comparison with experiment is challenging due to the limited availability of measured AE values for the graphene–gold interface. Specifically, Torres et al. found an AE of *E*_a_ = 7687.1 mJ/m^2^ = 48 eV/nm^2^ in an experiment exploring graphene-covered gold nanoparticles,^27^ whereas Li et al. found a pull-off force of ≈0.23 nN/nm^2^ = 1.44 eV/nm^3^ for gold–graphite interfaces.^23^ Notably, in order to obtain the AE measured by Torres et al., this force (even if assumed to remain constant) has to be applied along a distance of ≃33 nm, way beyond the interlayer interaction range. This indicates a discrepancy between the two experimentally measured values. We note that our calculated AE of about 0.5 eV/cell (375 mJ/m^2^) obtained for the trilayer gold–graphene interface model (Figure 3a) is lower than that found by Torres et al. while the corresponding pull-off force of 2.04 nN/nm^2^, evaluated from the first derivative of the SAIP AE curves, is larger than the experimental value of Li et al., suggesting that the SAIP provides values within the experimentally available range. A much better agreement is obtained between the SAIP predictions and previous computational results, such as an AE of 467 mJ/m^2^ calculated by Tesch et al. for graphene nanoflakes on Au(111)^15^ and 394 mJ/m^2^ recently calculated for the Cu(111)–graphene interface^50^ that is also dominated by vdW interactions.

**Figure 4:**
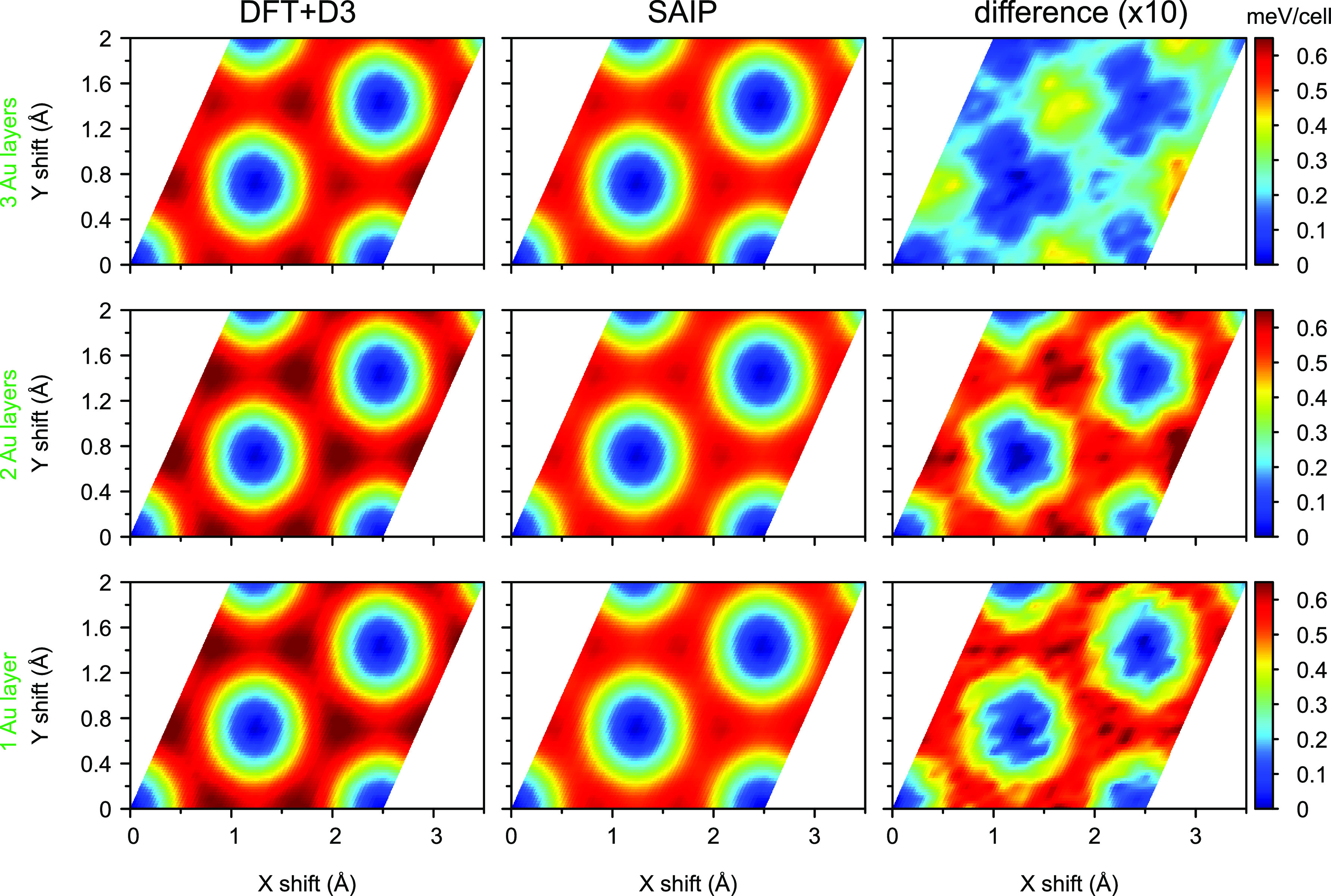
Graphene on gold: PES—sliding potential energy surface of the graphene–gold heterostructure, calculated at an interface separation of 3.5 Å. The upper, middle, and bottom rows are for trilayer, bilayer, and single layer gold, respectively. The left, center, and right columns present the PES calculated using DFT+D3, SAIP, and their difference, respectively. In the latter, the differences are magnified ×10 to clearly present the fine features. For better visibility, a 4 × 4 linear interpolation has been applied to all the PES maps. The minimum of the PES was shifted to zero for clarity.

The sliding PESs calculated using the SAIP parametrization show good agreement with the reference DFT+D3 data (see Figure 4), as well, with an error of 7.8, 11, and 10% for the PES corrugation of the trilayer, bilayer, and single layer gold, respectively, and corresponding average deviations of 0.010, 0.020, and 0.044 meV/cell, respectively. When comparing the AE of the different stacking modes considered (see Figure 3a), one finds that the maximum difference is about 0.5 meV/cell. This value is related to the CE as shown in Figure 4. We note that the CE is of the order of 0.075 meV per C-atom, indicating an exceptionally lubric contact.^22^ We would like to note that we obtain CE and AE values using energy differences of nearly identical systems, thus we expect a very high numerical accuracy. Furthermore, in the case of rigid contacts considered herein, such small CE ensues from the approximation employed to describe the lattice mismatch between gold and graphene. A truly incommensurate lattice mismatch, only obtainable in the thermodynamic limit of an infinite supercell size, would in fact yield a vanishing CE regardless of the potential parameters.

An LJ fitting of the DFT+D3 tri-layer gold–graphene PES resulted in ϵ = 14.86 meV and σ = 3.8 Å, in agreement with previous semi-empirical LJ parameterizations used to reproduce experimental results of gold cluster diffusion on graphene.^1,6^ Notably, an LJ fitting of the AE curves results in substantially different values, ϵ = 8.50 meV and σ = 3.37 Å, confirming that the isotropic LJ description is incapable of simultaneously describing the binding and sliding physics of vdW interfaces.

### 4.2 Benzene on Gold

**Figure 5:**
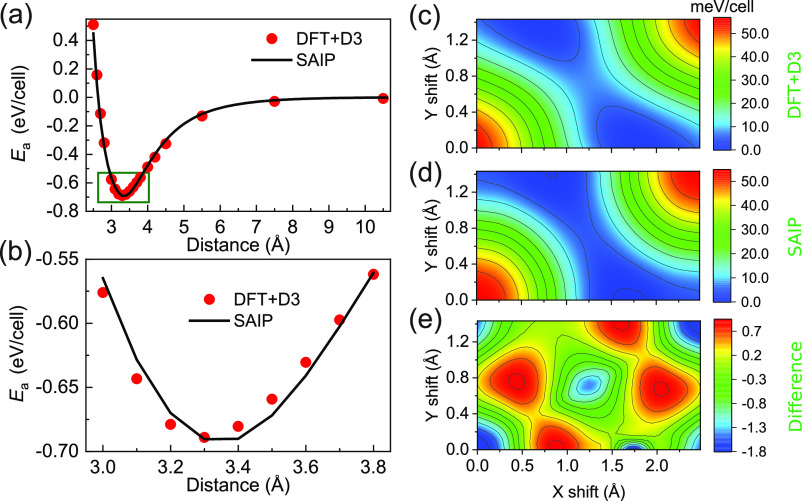
Benzene on gold—(a) AE curve, and (b) zoom-in around its minimum, of a benzene molecule residing over a trilayer gold surface at the configuration depicted in Figure 2c,d. Symbols and lines represent DFT+D3 reference data and SAIP results, respectively. The corresponding sliding PESs calculated using (c) DFT+D3 and (d) SAIP, and (e) their difference are obtained at a benzene–gold separation of 3.3 Å. For better visibility, isoenergetic contour lines are superposed on the PES maps, which were smoothened by a 8 × 8 linear interpolation. The minimum of the PES was shifted to zero for clarity.

Going beyond the periodic interface, we next consider the case of a benzene molecule residing atop a gold surface. Figure 5 reports the AE curves (Figure 5a,b) and sliding PES (Figure 5c–e) of the benzene/Au(111) heterostructure obtained using DFT+D3 and the SAIP using the parameters provided in Table 1. Good agreement between the DFT and SAIP results is found for both the AE curve and the sliding PES, with average deviations of 28.0 meV/cell (∼4% of the AE) and 0.73 meV/cell (∼1.4% of the CE) for the AE curve and the PES, respectively, and a deviation of 1.7% in the overall PES corrugation. The obtained AE value of 0.689 eV is in good agreement with a reference experimental value of ∼0.64 eV at finite temperature.^51^

We note that SAIP parameterization yields a smaller Au–H *C*_6_ coefficient and a larger *d* value than those of the corresponding Au–C parameters, suggesting a weaker and shorter-range dispersion term for the former. Furthermore, the Au–H anisotropic repulsive term (eq 5) is considerably weaker than the corresponding Au–C term. This results from the tenfold larger γ parameter of the former (see Table 1) that yields  for any reasonable value of ρ_*ij*_ in eq 5. Together with the fact that ϵ ≃ 2|*C*| in the Au–H case (see Table 1), the square brackets appearing in eq 5 are small in magnitude and quite insensitive to the value of the lateral interatomic distance. This indicates that the Au–H interaction has a minor effect on the lateral shear motion and that most of the lateral forces originate from Au–C interaction. Therefore, we expect that the present parametrization should hold for other planar benzenoid systems.

### 4.3 C_60_ on Gold

**Figure 6:**
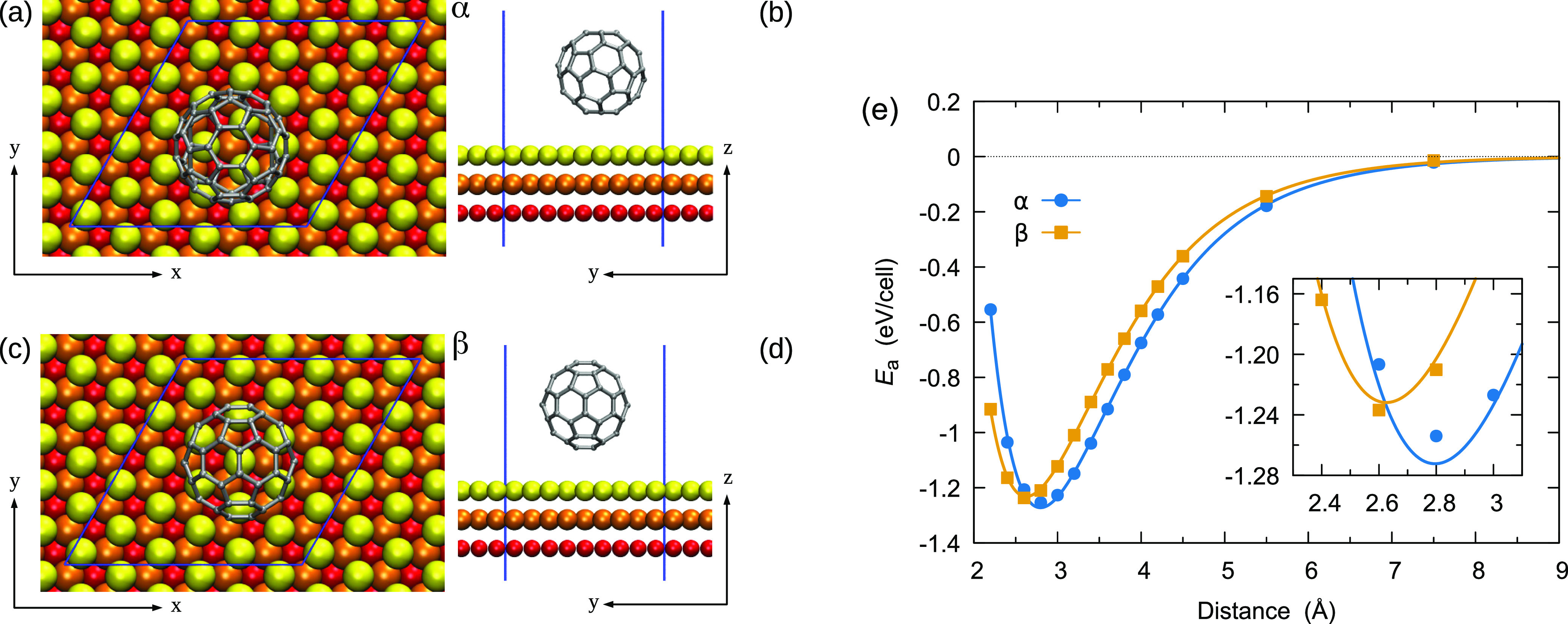
C_60_ on gold—(a) top and (b) side views of the C_60_/Au(111) model system in the α configuration. (c) Top and (d) side views of the C_60_/Au(111) model system in the β configuration. Carbon atoms are depicted in gray, and first, second, and third Au layers are colored in yellow, orange, and red, respectively. Blue lines outline the primitive cell. (e) AE curves for the α and β configurations, with symbols corresponding to the reference DFT+D3 data and solid lines to SAIP results obtained using the parameters appearing in Table 2.

Finally, we challenge our developed SAIP against extremely bent graphitic systems. In this case, one might expect significant deviations of the interfacial energy profiles from the ideally flat graphene case discussed above. In particular, it is known that mechanical bending can alter the reactivity of graphene sheets^52^ and introduce additional interfacial effects, such as curvature-induced structural lubricity.^53^ Since the latter effects should mainly depend on the structural properties (geometry) of the adjacent surfaces, they should be captured by the SAIP with the parameterization presented in Table 1. However, accounting for the different reaction energies of the curved graphene requires a full quantum-mechanical treatment and thus a reparameterization of the SAIP for the curved system. To demonstrate this, we considered the case of a C_60_ fullerene physisorbed over an Au(111) surface in two different orientations, marked as α and β, as depicted in Figure 6a–d. By performing DFT+D3 calculations at different separations from the Au(111) surface, we obtained the adhesion curves, reported in Figure 6e, showing adhesion energies of 1.2545 and 1.2373 eV for the α and β configurations, respectively. On average, the C_60_ AE is larger than that of benzene by about 80%, thus supporting the need to provide a separate parameterization for curved systems. This is in agreement with previous calculations indicating an increased reaction energy for nanotubes of a smaller curvature radius.^52^ The increased surface reactivity is manifested also in the equilibrium distance from the gold surface, which reduces from 3.48 Å for flat graphene to 2.6 and 2.8 Å for C_60_ at the α and β configurations, respectively (see Figure 6e). The simultaneous fitting of the two adhesion curves for C_60_ produced a distinct set of SAIP parameters, reported in Table 2. In comparison with the graphene/Au(111) interface, the larger *C*_6_ and smaller *d* parameters indicate a stronger and longer range vdW dispersion term. Nonetheless, the anisotropic term reduces significantly, mostly due to a ϵ ≃ 2|*C*| balance (see discussion above), indicating that the Au–C interaction is weakly dependent on shear motion in this highly curved system and that the sliding energy surface corrugation is expected to be very small. Similar considerations could be made in the case of very small-diameter carbon nanotubes (CNTs) on gold, for which Table 2 could apply. However, the SAIP parameters should approach those of Table 1 as the CNT diameter increases. The possibility of including such parameter transition in a revised SAIP functional form capable of describing general CNT–gold assemblies^54^ is presently under investigation.

**Table 2:** C_60_ Parameters—List of SAIP (Equations 1, 3, and 5) Parameters for the C_60_–Gold Interaction

atom pair	α	β (Å)	γ (Å)	ϵ (meV)	*C* (meV)	*d*	*s*_R_	*r*_eff_ (Å)	*C*_6_ (eV Å^6^)
Au–C	11.25629	3.42336	5.37200	0.186756	–0.093183	8.89407	1.098183	3.650286	90.433089

## 5 Conclusions

The results presented above indicate that the proposed semi-anisotropic interface potential (SAIP) is able to accurately reproduce the energetics of graphene–gold, benzene–gold, and C_60_–gold interactions, as obtained from DFT+D3 calculations. While the functional form of the SAIP is suitable to treat many interfaces between graphitic systems and gold surfaces, system specific parametrizations of the SAIP are recommended in order to obtain optimal accuracy. However, the presented potential is expected to describe the structural and dynamical response to external forces of a large number of prototypical systems, such as gold nanoclusters on graphite,^3,4^ graphene nanoribbons on Au(111),^7−9^ and C_60_ on Au(111),^55,56^ among others, with much improved accuracy with respect to previous classical models. Furthermore, our formulation can be generalized to describe a wide variety of interfaces between hexagonal 2D materials and bulk solids, such as MoS_2_/Au,^25^ h-BN/Au,^24,26^ or graphene/Ag.^15^ This, in turn, will considerably increase the scope of material interfaces that can be treated using reliable dedicated classical force-fields.
